# Rapid Annotation of Anonymous Sequences from Genome Projects Using Semantic Similarities and a Weighting Scheme in Gene Ontology

**DOI:** 10.1371/journal.pone.0004619

**Published:** 2009-02-27

**Authors:** Paolo Fontana, Alessandro Cestaro, Riccardo Velasco, Elide Formentin, Stefano Toppo

**Affiliations:** 1 FEM-IASMA Research Center, San Michele all'Adige (TN), Italy; 2 Department of Biology, University of Padova, Padova, Italy; 3 Department of Biological Chemistry, University of Padova, Padova, Italy; University of Pennsylvania School of Medicine, United States of America

## Abstract

**Background:**

Large-scale sequencing projects have now become routine lab practice and this has led to the development of a new generation of tools involving function prediction methods, bringing the latter back to the fore. The advent of Gene Ontology, with its structured vocabulary and paradigm, has provided computational biologists with an appropriate means for this task.

**Methodology:**

We present here a novel method called ARGOT (Annotation Retrieval of Gene Ontology Terms) that is able to process quickly thousands of sequences for functional inference. The tool exploits for the first time an integrated approach which combines clustering of GO terms, based on their semantic similarities, with a weighting scheme which assesses retrieved hits sharing a certain number of biological features with the sequence to be annotated. These hits may be obtained by different methods and in this work we have based ARGOT processing on BLAST results.

**Conclusions:**

The extensive benchmark involved 10,000 protein sequences, the complete *S. cerevisiae* genome and a small subset of proteins for purposes of comparison with other available tools. The algorithm was proven to outperform existing methods and to be suitable for function prediction of single proteins due to its high degree of sensitivity, specificity and coverage.

## Introduction

The amount of data available in public databases has reached an unprecedented complexity which is not easily manageable by users. 789 genomes have been completed and over 1,600 are in progress assembly (as of November 2008). This means that thousands of raw sequences are readily obtainable, but the challenge now [1] is to assign them a putative function and to keep the annotation up-to-date [2]. The results are difficult to interpret, especially when the retrieved hits share low sequence identity with the starting query or when restricted local alignments identify a single domain in multi-domain protein hits or, finally, when the updating state of protein hits is incomplete and even contradictory making functional transfer difficult [3].

The definition of protein function itself is elusive and ambiguous as it depends on i) context: where the protein acts and its behavior in particular conditions; ii) scale: the level at which functional assignment is reported, namely molecular or cellular and organismal; iii) time: when and for how long a certain protein operates in the cell's life-span [3], [4].

Against this background, the Gene Ontology (GO) consortium has developed a successful solution that may be considered the gold standard in functional classification [5], [6]. It uses a structured controlled vocabulary organized in a hierarchical Directed Acyclic Graph (DAG) that has two important characteristics: it has become an acknowledged and widely used framework for functional annotation and it is designed to be easily exploited by computational methods [7]. However, some questions still remain unanswered as the Gene Ontology structure provides a static representation of biological function but does not account for the dynamics of protein expression in the metabolic and structural pathways of the cell's life cycle [6]. Currently, sequences deposited in public databases are automatically reviewed with increasing accuracy and annotation coverage is nearly 60% of the total proteins (as of November 2008). On the other hand, manual curation of protein function and knowledge transfer from both experimental data and the literature still lag behind, even though increasing interest is pushing international efforts to close the gap as soon as possible.

Before the advent of the Gene Ontology, different independent studies provided conflicting results: varying estimates have been proposed to infer function from sequence identity showing the difficulties in reaching a shared agreement. The thresholds for accurate prediction oscillate from a lower bound of 40% [8] sequence identity to the more stringent criterion of 60–70% [9], [10], but the latter estimate suggests a cut-off of 50% [11]. These differences reflect the intrinsic difficulties with functional inference from simple evaluation of sequence identity and show an urgent need for a critical assessment of acknowledged standards in prediction methods [12], [13]. In recent years, different approaches have been proposed to improve function prediction performance. King and colleagues [14] were among the first to demonstrate the potential for gaining new information from machine learning algorithms and GO annotation patterns. Lord and colleagues introduced the use of semantic similarity to investigate the relationship between sequence and annotation [15], [16]. Semantic similarity among concepts has been used extensively in natural language processing and seems to be perfectly suited to Gene Ontology. It is, in fact, a manageable metric which reflects the closeness or distance between the concepts, corresponding to weak or strong biological relationships [15]. Recently, Tao and colleagues [17] extended this idea proposed by Lord in information retrieval and based their method on measurement of the distances between clusters of existing GO annotations which are shared by sequences. This approach has proven to be effective in extending gene function information and to have higher recall than previously published methods.

Other approaches rely on text mining and information extraction systems applied to the biological domain, such as the BioCreAtIvE initiative [18]. These methods still perform poorly as they attempt to interpret free text extracted from scientific literature, where the same concept may be described in different ways, whereas in GO each concept is unequivocally coded. It is undoubtedly true that one of the main limits of published strategies for function prediction is the source of information. Using a simple similarity search like BLAST [19] to annotate unknown sequences may be more successful in terms of coverage than querying databases of structural and sequence features using complex algorithms; this is due to the discrepancy between primary databanks, containing millions of sequences, and specialized databanks, containing a few thousand 3D structures, protein domains and functional patterns. In support of this idea, Jones and colleagues [2] suggest that the first returned BLAST matching hit is a fairly good strategy for annotating novel sequences, arguing that new methods should at least be able to outperform this approach. On the other hand, BLAST search alone is not sufficient to infer function as previously demonstrated [8], [9], [10], [11]. We here describe a method that partially overcomes these limitations by implementing semantic similarities among GO terms and an appropriate weighting scheme. To satisfy all of these needs, we have developed a fast method called ARGOT (Annotation Retrieval of Gene Ontology Terms) that is able to interpret the “GO dialect” and analyze hundreds of sequences very rapidly. The algorithm works on an acquired source of information, usually a list of ranked hits whose scores reflect biological similarity to the starting query. To our knowledge, ARGOT is the first example of a tool implementing semantic similarity and weighting schemes based on scores extracted from, for instance, the well-established sequence similarity search. GOtcha, developed by Martin and colleagues [20], may be considered the first automatic tool for function prediction which takes advantage of a weighting scheme based on term-specific probability measures of confidence, but it does not consider the semantic similarities among retrieved terms. Indeed, semantic similarity is generally used as a metric to evaluate common and distant features among sequences and their annotated functions [21] but has never been effectively employed in the process of annotating novel sequences. We tested ARGOT using the common BLAST similarity search against the primary repository UniProtKB and assessed it over two large benchmark test sets. The first consisted of 10,000 randomly chosen proteins extracted from UniProtKB, while the second comprised the well-studied *S. cerevisiae* genome for which in-depth functional knowledge is available.

Using BLAST results allowed us to compare the ARGOT method with other available tools, in particular Blast2GO [22], using the same file source and the TOPBLAST strategy [20], which is based on the functional transfer of annotations carried by first significant BLAST hits.

A further small and curated dataset of 28 targets was evaluated and compared with the following function prediction methods available on the web: JAFA [23], Blast2GO [22], GOtcha [20], PhyDBAC [24], GOblet [25], PFP [26], InterProScan [27].

We demonstrate here that by using a simple similarity search valuable information with a high degree of confidence and high coverage can be obtained, even at low sequence identity, thanks to a strategy that carefully equates semantic similarity with shared and ranked biological features. The tool has already been employed in the annotation of over 29,000 predicted gene sequences from the large scale *Vitis vinifera* sequencing project [28] and has been used to annotate a small subset of over 500 sequences from a cDNA-AFLP sequencing project in *Medicago truncatula* [29]. Manual validation of the annotations acquired for this small subset of sequences of *Medicago truncatula* confirmed the high quality of the functional inference processing tool.

ARGOT is available free to academic users at the following URL: http://genomics.research.iasma.it/argot/index.html.

## Materials and Methods

### Trimming the GO graph

**Figure 1:**
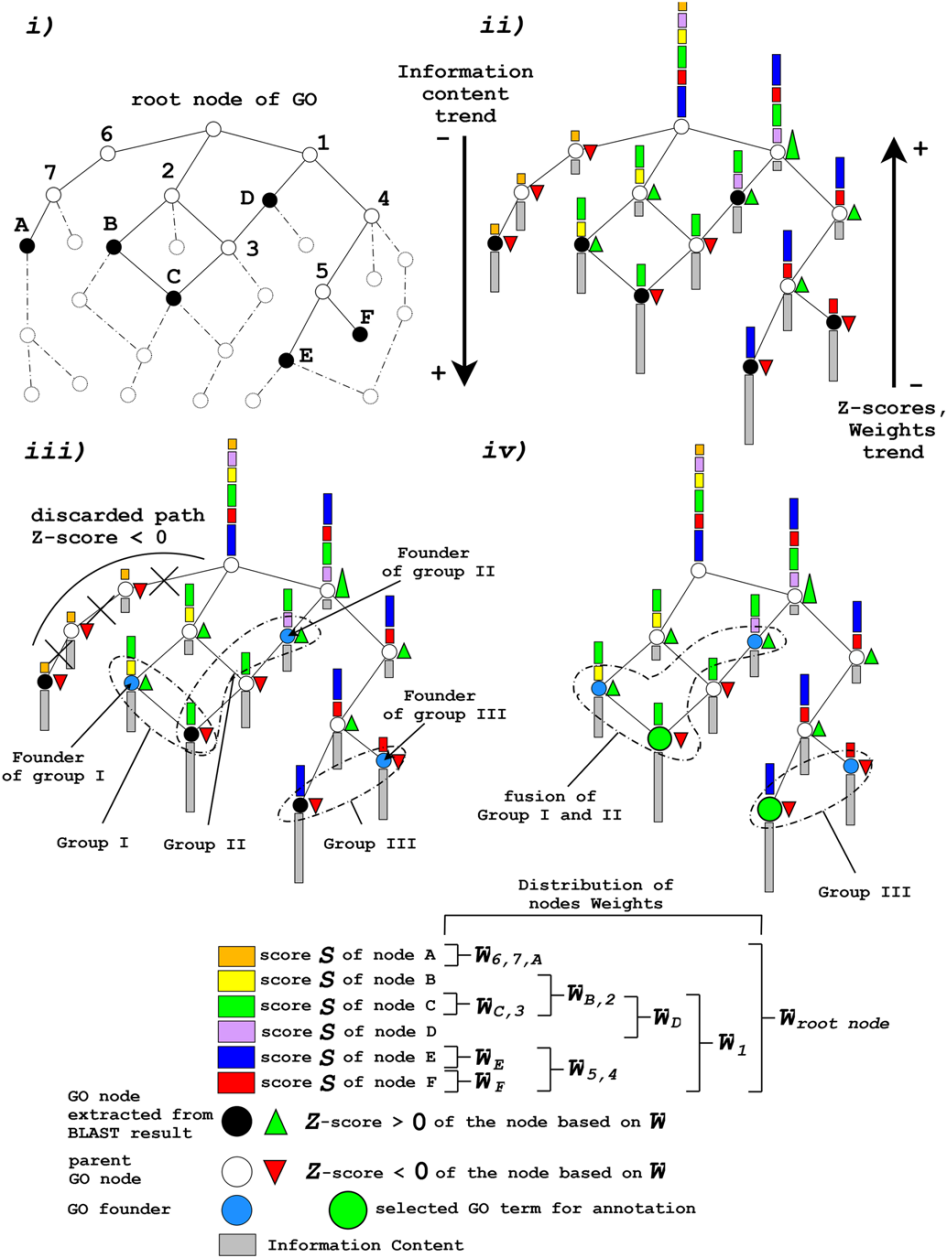
Schematic representation of the ARGOT algorithm. The sections of the “materials and methods” where the different steps of the algorithm are explained in details, are enclosed in quotation marks. The first step *i)* “Trimming the GO graph” involves the trimming of the GO graph to obtain a slim containing only the putative GO hits extracted in order to annotate the query protein (black circles). In the second step *ii)* “Weighting the GO nodes” and “GO Information content”, the algorithm calculates the Information Content (*IC*) (gray bars) of the nodes in the graph and their cumulative weights (colored bars) derived from the BLAST scores. In the third step *iii)* “Grouping by means of semantic similarity” and “Choosing the most probable paths in the “initial trimmed GO graph””, GO nodes are clustered into groups having a given semantic similarity using the Lin formula; GO terms which populate isolated branches of the graph are discarded on the basis of their Z-score (red and green triangles) applied to the node weights. In the last step *iv)* “Grouping by means of semantic similarity” ARGOT tries to merge similar clusters on the basis of semantic distance calculated among the groups' founders (azure circles) using less stringent cut-offs. GO terms with the highest *IC* (green big circles) are chosen as representatives of the clusters obtained.

The functional inference of a query sequence is performed on a starting list of scored and ranked GO terms (see Fig. 1). In principle, the ARGOT algorithm is not dependent on the method used to obtain this list. In the present work we used the BLAST searching tool, as it is the most commonly used method for annotating biological sequences. The first step of the algorithm involves the extraction of the GO terms from those BLAST hits which are annotated in Gene Ontology. Each GO term inherits the BLAST e-value score of the corresponding hit from which it has been extracted. If multiple hits share the same GO term, the sum of the logarithms of their e-value scores is calculated as explained in the next section “weighting the GO nodes”. Once GO hits and their corresponding scores are obtained, they are processed in order to reconstruct all of the possible paths leading to the root node; the rest of the GO nodes that does not belong to the reconstructed paths are discarded and we finally obtain the “initial trimmed GO graph” (see Fig. 1-*i*). The user can set the number of hits ARGOT has to analyze for annotation (the default is 50).

### Weighting the GO nodes

Once the “initial trimmed GO graph” has been obtained, each node is weighted according to the BLAST e-values. The corrected weights are reconstructed starting from the leaves of the graph up to the root following a non-redundant and cumulative strategy (see Fig. 1-*ii*). Cumulative means that the weight of the parent node is the sum of the weights of its child nodes. During the exhaustive reconstruction of the multiple paths leading to the root of the graph, some parent nodes may be visited many times. Non-redundant means that the score of a child node is added only once to the weights of the parent nodes during this process. The weight W is calculated as follows:
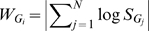
Where 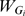 is the weight of the node 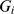, 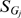 is the score of the node and 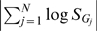 is the absolute value of the sum of the child nodes' scores. These scores are extracted from the results of BLAST as described in “Trimming the GO graph” section. The weighting strategy is always non-redundant and cumulative. What may change is how the original extracted scores are converted and normalized. In this case, we have used the logarithm of the BLAST e-values and the absolute value of their sum. As a result of this additive strategy, only the most generic annotation terms, those near the root, show the highest scores at the expense of carrying less information. To tackle this behavior a different measure based on the Information Content (IC) was used to obtain an acceptable trade-off between detailed knowledge and statistical significance (see the section “GO Information content”).

### GO Information content

The standard definition of information content of a term in a taxonomy, as proposed by Resnik in 1999 [30], is quantified as the negative log likelihood as follows:
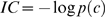
Where *IC* is the Information Content and *p(c)* is the relative frequency of all of the descendants of the GO term *c* that are extracted from the GOA database.
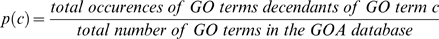
Depending on the GOA database release used, the *IC* value changes as a consequence of continuous enhancements and updates of both the protein annotations and the GO graph structure.

The formula expresses the notion that informativeness decreases as the frequency of a term increases. The probability of any concept appearing in the taxonomy depends on the sum of the probabilities of the concepts it subsumes and, consequently, the nearer the term is to the root the more frequent and the less informative the concept is (see Fig. 1-*ii*).

### Choosing the most probable paths in the “initial trimmed GO graph”

In the “initial trimmed GO graph” some terms may belong to hits that are not functionally related to the original query. Usually the GO terms of these hits are found in isolated paths within the graph and consequently have low weights. This typically occurs when false positive hits sharing low sequence similarity with the query are considered for functional annotation transfer. To avoid the subsequent computation of these GO terms, which may be unrelated to the query sequence, the Z-score cut-off is applied on the node weights as follows:
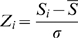
Where 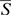 is the average calculated as the score of the root node divided by the total number of the nodes that compose the “initial trimmed GO graph”, 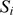 is the score of node *i* and σ is the standard deviation assuming a Gaussian distribution of the weights (see Fig. 1-*ii*). The path is chosen if, starting from the leaves, its Z-score becomes positive before reaching the root which, by default, has a positive value as it represents the sum of all the node weights (see Fig. 1-*iii*). This approach allows paths that are statistically significant to be discriminated from those that are not. Low scoring nodes (see Fig. 1-*iii*) do not contribute to the general annotation path and reduce the computational efficiency. At the end, these nodes and their paths are discarded and the initial trimmed GO graph is reduced in size. We here refer to the resulting graph as the “final trimmed GO graph”

### Grouping by means of semantic similarity

Once the “final trimmed GO graph” has been obtained, the remaining GO nodes are grouped according to their semantic similarity (see Fig. 1-*iii*,1-*iv*). This approach has already been applied in natural language processing and taxonomies providing a manageable metric with which to link different terms carrying similar information. There are many different methods for calculating the shared information between two terms [30], [31], [32], but we obtained the best results using Lin's formula [33]. This formula has the advantage of reporting a normalized value between 0 and 1 and has already been proven to outperform other algorithms [17] even though it may be affected by shallow annotations [34], [35]. Lin's formula is defined as:
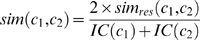
Where
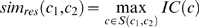


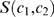
 are the common subsumers of 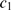 and 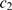 terms and 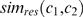 is the one with the highest *IC* as previously described in the section “GO Information content”. In other words, the algorithm finds the nearest parent node that is shared by the two terms whose semantic distance is to be calculated.

This quantifies the extent to which two concepts are related according to their position, which is highly dependent on the graph connections and density. This measure is much more effective and meaningful than absolute edge distance as it accounts for the real relationships among concepts. For example, the two GO nodes GO:0043734 “pigmentation” and GO:0016032 “viral reproduction” have the root node GO:0008150 “biological process” as parent. These GO terms may be erroneously considered neighbors as their edge distance is 2 but their meaning is completely different. This difference appears immediately evident from a semantic similarity perspective, as the *IC* of the parent node is 0, being the root of the ontology, and consequently the semantic similarity is 0, as expected. The same edge distance exists between two similar GO terms: GO:0004602 “glutathione peroxidase activity”, which occurs 69 times over 168,919 gene products (as of March 2008 GO release), and GO:0047066 “phospholipid-hydroperoxide glutathione peroxidase activity”, which occurs 5 times. They both descend from a common ancestor GO:0004601 “peroxidase activity” with 409 occurrences, subsuming all the occurrences of its child nodes. In this case, semantic similarity calculated with Lin's formula is 0.66 as follows:
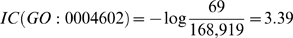


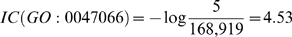


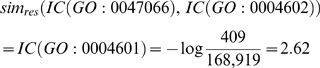


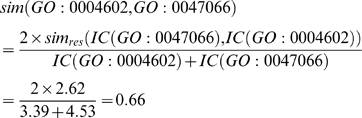
This reveals that the GO terms are strict neighbors. Comparing the two examples it is clear that edge distance is unsuitable and insufficiently discriminative for finding related concepts.

Semantic similarity is used to group GO terms that belong to the “final trimmed GO graph” containing the most probable starting paths and allows the number of similar GO terms in highly populated regions of the graph to be further reduced (see Fig. 1-*iii*,-*iv*). The algorithm attempts to group recursively the GO terms that have been retrieved by the BLAST hits ***R*** and belong to the “final trimmed GO graph” with strict semantic similarity of over 0.7. Those GO nodes that are part of the different paths leading to the root of the graph but that have not been found in the annotation of the BLAST hits, are not considered in this calculation.. A matrix distance is computed by performing an all vs. all comparison of the *IC* scores of the GO terms retrieved from ***R*** using the Lin's formula and if two terms satisfy the cut-off they are merged into the same group (see Fig. 1-*iii*):
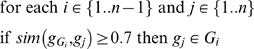


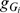
 is the initiator node of the group 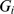. The initiator, 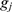 is another GO node and *n* is the total number of GO terms extracted from ***R*** and present in the “final trimmed GO graph”. At the beginning of the clustering process any GO term is a potential initiator node and is considered the founder of a forming cluster: the GO terms sharing a semantic similarity of 0.7 are added. After the first clustering step a second and less stringent threshold of 0.6 is applied to further merge acquired groups that have at least one GO term in common. Only the initiator nodes or founders 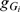 are involved in this process (see Fig. 1-*iv*). The grouping strategy has the effect of gathering together semantically similar GO terms thus actually reducing the search space as only one or few representatives per group are chosen according to their scores, described in the next section, and *IC*.

### InC, AC, and TS scores

First of all, the GO hits that belong to the obtained clusters in the “final trimmed GO graph” are ranked on the basis of three different statistical scores: Internal Confidence (InC), Absolute Confidence (AC) and Total Score (TS). GO terms with the highest scores and IC are chosen (see the section “GO Information content”). The InC and AC are normalized scores whose values lie in the 0–1 interval; they have been specifically designed to assess the statistical significance of the retrieved hits and both are based on node weights divided either by the root node weight (InC) or by the maximal theoretical weight (AC). The TS score is derived from the InC multiplied by the Z-score of the node under consideration and is not normalized as it starts from 0 and has no upper bound.
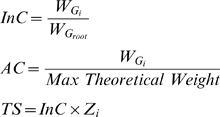
Where 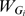 is the weight of the node 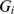, 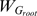 is the weight of the root node (see the section “Weighting the GO nodes”), *Max Theoretical Weight* is the maximal score the query sequence can get based on the algorithm used to investigate the database, 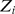 is the Z-score of node *i*. For the BLAST searching algorithm the *Max Theoretical Weight* has been set to 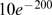 corresponding to a highly significant hit based on the e-value score.

The user can choose among TS, AC, and InC indexes and set the cutoff for ranking and selecting the GO terms. GO terms not satisfying the threshold are discarded. In addition, the user can choose the number of GO terms that can be extracted from each cluster and these are called the representatives of the cluster. These representatives are chosen on the basis of their highest information content (IC) rather than their score based on the chosen index among TS, AC, or InC. This strategy allows the algorithm to select the most informative representatives of the clusters rather than those with the highest AC, TS, or InC scores.

### Algorithm implementation

ARGOT has been implemented in JAVA and accompanying scripts are provided to set up the tool on a local computer running Linux OS and MySQL database. An example script running BLAST and ARGOT is supplied to perform batch processes of protein sequence annotation. The tool is available free to academic users and can be downloaded from the following URL: http://genomics.research.iasma.it/argot/index.html.

### Construction of the test sets RES and YEAST

**Figure 2:**
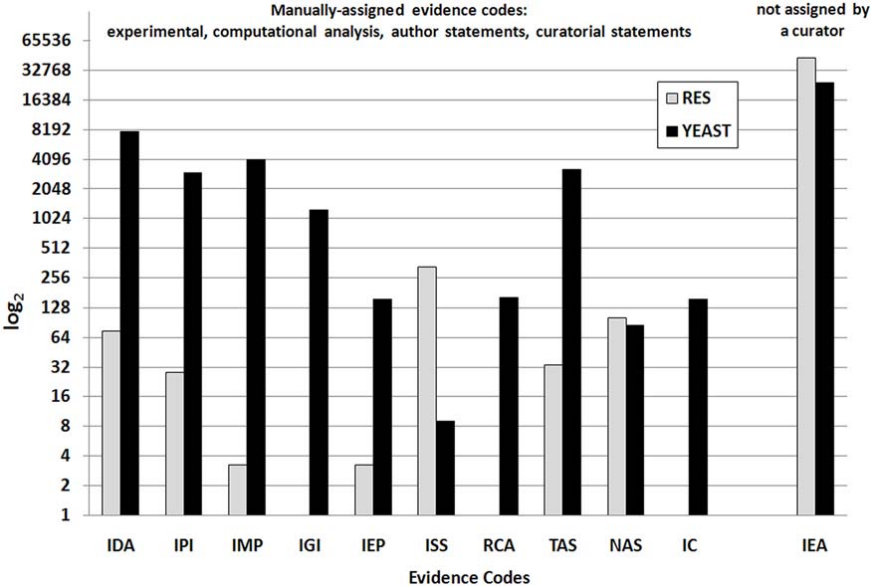
Distribution of GO evidence codes in the test sets RES and YEAST. The evidence codes are as follows: Inferred from Direct Assay (IDA), Inferred from Physical Interaction (IPI), Inferred from Mutant Phenotype (IMP), Inferred from Genetic Interaction (IGI), Inferred from Expression Pattern (IEP), Inferred from Sequence or Structural Similarity (ISS), inferred from Reviewed Computational Analysis (RCA), Traceable Author Statement (TAS), Non-traceable Author Statement (NAS), Inferred by Curator (IC), Inferred from Electronic Annotation (IEA). The y-axis reports the log_2_ value of the distribution in each category. For further information on evidence codes, please see the GO annotation guide on the GO Home Page (http://www.geneontology.org/GO.evidence.shtml).

To test the efficacy of the ARGOT method, 10,000 sequences were randomly extracted from the GOA database (release June 2007) with at least one GO term associated to perform the blind test. No restrictions were applied in order to simulate a real random test case. This test set was called RES (Randomly Extracted Sequences) (see Fig. 2).

The method was further assessed by simulating the annotation of a whole genome with a high level of annotation accuracy, such as *S. cerevisiae* [36] (see Fig. 2 showing the evidence code distribution), extracted from UniProtKB (release 12.0, June 2007). This test set was called YEAST.

### Construction of a small reference test set

Comparison with other tools, which are not available for downloading and hence with which it is not possible to perform an extensive benchmark, was assessed on a well-annotated small subset of sequences extracted from Uniref50 (release 13.0, March 2008). The sequences were chosen from those having at least one GO term in their molecular function ontology and with at least one non-IEA evidence code reported in the GOA database (meaning that experimental data are available). Proteins carrying only general and shallow GO terms in the graph, such as “protein binding” (GO:0005155), were discarded. Further filtering was applied on the basis of sequence similarity vs. UniProtKB (release 13.0, March 2008). To test effectively the performance of these methods in retrieving the right annotation from hits with low sequence identities, only proteins having at least 50 BLAST hits and with the first hit in the range of 25–50% sequence identity, were considered for testing. Further manual checks and pre-processing of original annotations were carried out in order to confirm protein functions. Selected sequences were then submitted to the following servers: JAFA, GOtcha, PhyDBAC, GOblet, Blast2GO, PFP, and InterProScan.

### BLAST searches and TOPBLAST

The sequences of the two test sets were searched using BLAST against UniProtKB (version 12.0, June 2007) with default values. Those of the test set RES had been eliminated from the UniProtKB databank before BLASTing and restrictions were also applied to the YEAST test set in that all of the sequences from Fungi were discarded.

The TOPBLAST [20] strategy is based on direct assignment of GO terms extracted from the first top hits of the BLAST output. These GO terms are used for functional transfer and annotation. Sequence similarity is used as a cut-off to assess positive or negative prediction (see the section “Statistical analysis”).

### Blast2GO

The same BLAST results produced for ARGOT annotation were analyzed with Blast2GO [22], which was the only tool freely available for local installation at the time of benchmarking. This tool predicts function from BLAST results and is suitable for direct comparison with ARGOT as it uses the same file source.

### Statistical analysis

The performances of the methods were assessed using ROC (Receiver Operating Characteristic) curves. The varying discrimination thresholds for ARGOT were the three scores AC, InC and TS, whereas for TOPBLAST sequence identity was used, as explained below. To avoid undermining the accuracy of the statistical assessment, the predicted GO terms were treated as correct if and only if they were identical to the original annotations of the test set. Using different permitted distances between the correct and the predicted GO terms was not considered rigorous nor biologically appropriate, as shown elsewhere [37]. The quantities were calculated as follows:

TP (True Positive): the method assigned the right GO term (good prediction)

FP (False Positive): the method assigned the wrong GO term (bad prediction)

TN (True Negative): the method did not assign the wrong GO term (good prediction)

FN (False Negative): the method did not assign the right GO term (bad prediction)

“Right GO term” refers to the real annotations of the test sets and “wrong GO term” refers to errors.

**Figure 3:**
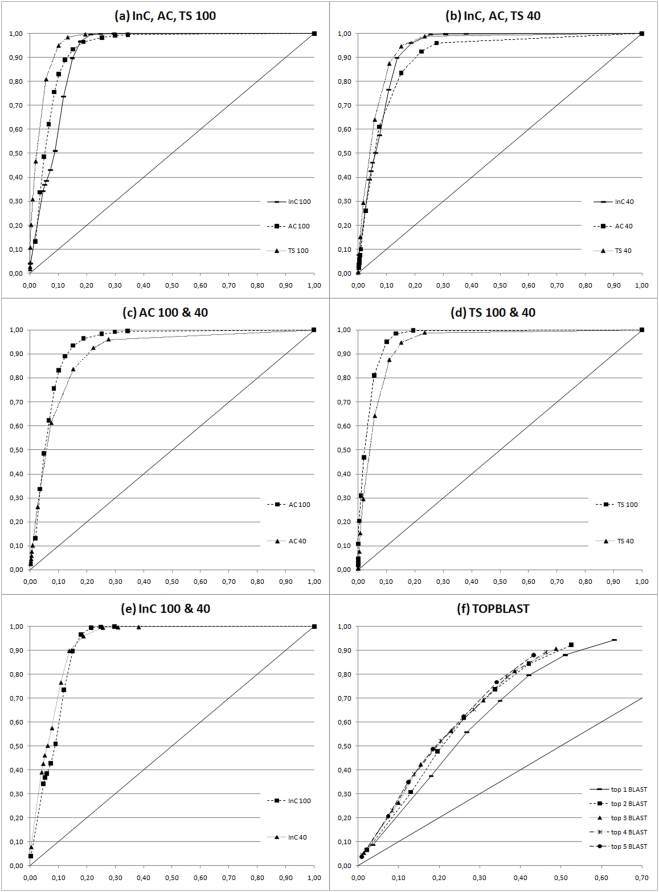
ROC plots of the benchmark test set RES. In (a) the results of InC, AC and TS scores are reported for hits under 100% sequence identity (ROC 100 plots). In (b) the performances of the three indexes are reported for low sequence similarity hits below 40% identity (ROC 40 plots). In (c), (d), and (e) the AC, TS, and InC scores are shown respectively, with comparisons of their trends at low (ROC 40 plots) and high (ROC 100 plots) sequence similarity. In (f) the annotations of up to the first top five BLAST hits are evaluated (TOPBLAST). See M&M for further details.

**Figure 4:**
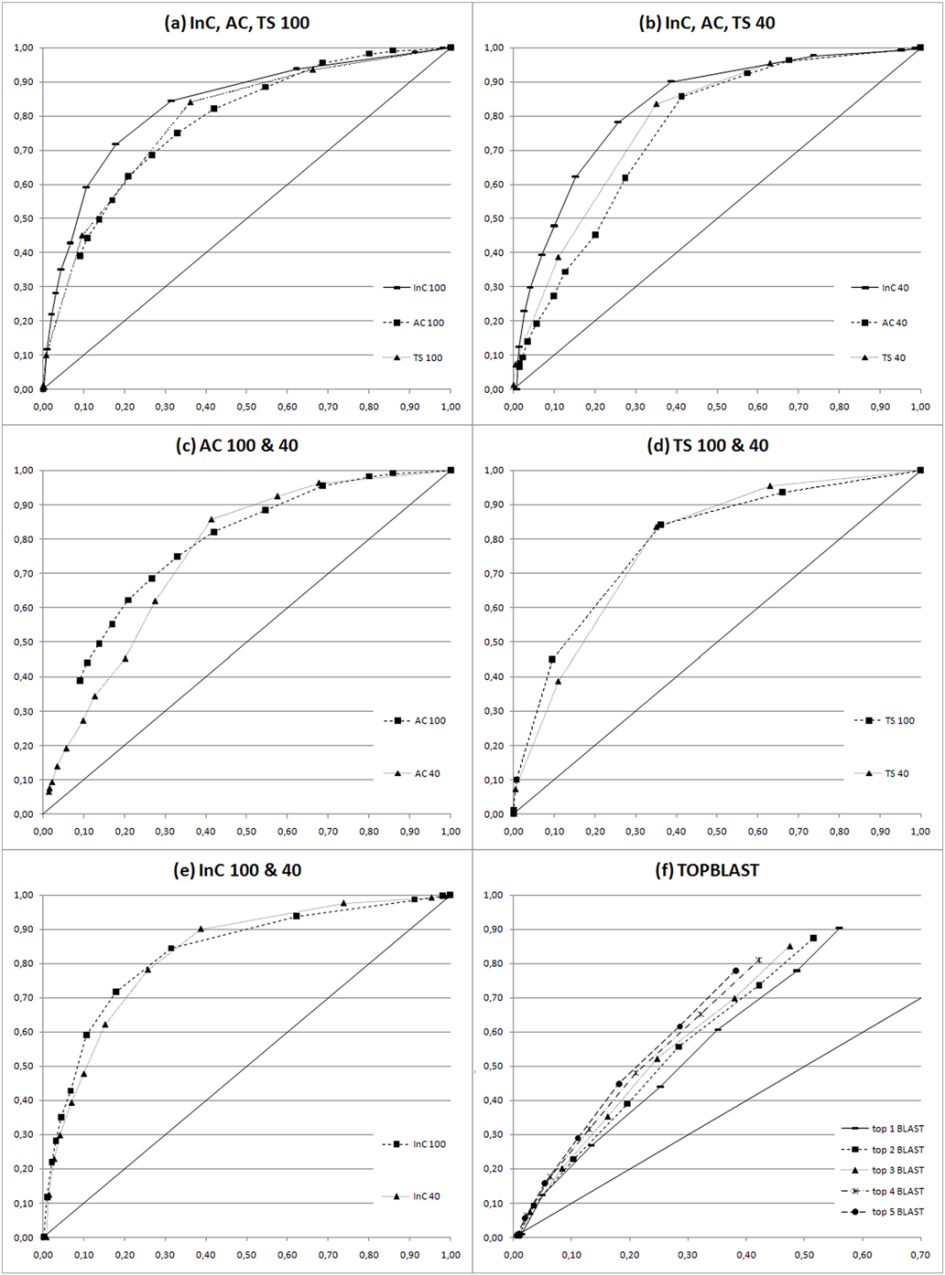
ROC plots of the benchmark test set YEAST. In (a) the results of InC, AC and TS scores are reported for hits under 100% sequence identity (ROC 100 plots). In (b) the performances of the three indexes are reported for low sequence similarity hits below 40% identity (ROC 40 plots). In (c), (d), and (e) the AC, TS, and InC scores are shown respectively, with comparisons of their trends at low (ROC 40 plots) and high (ROC 100 plots) sequence similarity. In (f) the annotations of up to the first top five BLAST hits are evaluated (TOPBLAST). See M&M for further details.

In benchmarking the BLAST performance, we considered only the first BLAST hit in the “top 1 BLAST” class up to the first five BLAST hits in the “top 5 BLAST” class (see ROC curves in Fig. 3(f) and Fig. 4(f)) and we chose the sequence identity calculated by BLAST as the varying discrimination threshold. The interval investigated ranged from 100% down to 40% with decremental steps of 10% in sequence identity. A GO term was counted as a positive prediction (either TP or FP depending on the match with the real annotation) if it belonged to a hit found above the current cut-off and conversely a negative prediction if found below (either TN or FN). As expected, highly similar BLAST hits are assumed to belong to the same protein family and hence share the same GO terms. We did not eliminate this redundancy and automatically counted GO terms as many times as they occurred. We used this strategy because we observed that the same GO terms were found both above and below the chosen threshold causing problems for their correct evaluation. Blast2GO did not report any score and version 1.2, which we used locally at the time of benchmarking, was time consuming due to unpredictable stops; we therefore limited the comparison to 4,000 sequences and stopped the analysis after a one-month calculation. These problems have now been solved in the current Blast2GO release. To allow a fair comparison with Blast2GO we treated ARGOT results as if they had the same score.

Specificity (TN/(TN+FP)) and sensitivity (TP/(TP+FN)) were calculated and reported in the x-axis (1 - specificity) and y-axis (sensitivity) of the ROC plots respectively. The ROC curve shows the extent to which the method is able to obtain as many positive results (increase of sensitivity) as possible at the expense of increasing false positive predictions (decrease of specificity). To assess the positive and negative predictive rate both the positive predictive value (PPV = TP/(TP+FP)) and the negative predictive value (NPV = TN/(TN+FN)) were calculated at those thresholds that were chosen as having the best trade-off between positive and negative predictions after the ROC curve analysis. These measures are more intuitive as PPV accounts for the proportion of positive results which are really true, whereas NPV accounts for the proportion of negative results which are really false. The accuracy parameter (ACC = (TP+TN)/(TP+FP+TN+FN)), which accounts for the degree of closeness to the true result, was also calculated.

## Results

### Test set evaluation

Before starting the analyses, sequences belonging to the test set RES were clustered at different identity thresholds using CD-HIT [38] to confirm the unbiased composition, in terms of sequence identity, of the randomly extracted proteins (see Table 1). A further check was carried out to calculate the real coverage of unique GO terms represented in the test set compared to their total in the Gene Ontology graph (see Table 1). No quality checks were performed on the test set YEAST as it represents the proteome of an entire organism and the whole range of functional classes is guaranteed. We also calculated the distribution of the evidence codes that reflect the different types of associated functional descriptions. As expected, a greater percentage of curator reviewed annotations is present in the YEAST test set compared with the RES test set (see Fig. 2). This is because over 95% of the deposited proteins in UniProtKB are inferred from computational methods and not manually checked (IEA evidence code). This does not entail that IEA annotated proteins are less accurate than sequences with experimental evidence. Incomplete, too generic, or erroneous information may affect even well-studied proteins. For instance, a number of false positives may be found even in high throughput yeast two-hybrid assays with consequent erroneous functional assignments (IDA, IPI, or RCA evidence codes) in the protein interaction maps [39]. However, reliable progress has been made in the GOA consortium's automatic annotation pipeline and efforts have been made to limit the error rate in the annotation records [40]. This has motivated us not to discard IEA annotated proteins in the test sets nor in the databank.

**Table 1:** Some details of sequence and GO composition in the test set RES.

**GO coverage**	40.2%
**Cluster identity**	**Redundancy**
**90%**	4.15%
**80%**	7.00%
**70%**	11.10%
**60%**	16.50%
**50%**	23.30%

GO coverage represents the percentage of unique and different GO terms in the test set RES calculated over the whole Gene Ontology. The cluster identity accounts for the fraction of sequences, expressed as percent in the “redundancy” column, sharing different levels of sequence identity reported in the “cluster identity” column.

In addition, the UniProtKB database for BLAST searches has not been modified further and sequence redundancy has not been reduced. In fact, sequences belonging to the same protein family should carry the same functional features, but their annotations may vary from sequence to sequence depending on varying factors. One of these may be a certain asymmetry in the relationship between sequences and their GO terms due to the updating state of the databanks. A recent sequence submission may improve on and outdate previous annotations for only one member of a protein family, but the other members might not benefit from this acquisition. Consequently, reducing complexity and redundancy in sequence databases may negatively affect efficient functional inference. ARGOT has been specifically designed to annotate the starting query by finding the best trade-off between specificity of GO terms and sequence features or similarities to annotated hits.

### ROC plot evaluation

We assessed ARGOT's performance using ROC plots to test the behavior and robustness of the method over different thresholds of sequence identity. The assessment was carried out at GO term level as the proteins of the test sets contain an average of more than four GO terms each. For this reason, the performance of the method cannot be effectively evaluated at protein level as the assignment of an exact result over a wrong one is uncertain, whenever a mix of correct and incorrect predictions is recovered for every protein. In addition, the benchmark test was designed to retrieve the original annotations of the starting proteins in order to obtain the best unbiased view of the method's performance. Using semantic similarity or counting the minimum edge distance of two terms in the GO graph, would result in an overestimation of performance [2] and would not account for particular cases where near concepts in the graph may be biologically unrelated. At two edges distance, for instance, GO terms may share the same parent node but have different meanings, such as the GO identifiers GO:0004602 and GO:0004096 which correspond respectively to “glutathione peroxidase activity” and “catalase activity”. Though both proteins exert a “peroxidase activity” (common parent node GO:0004601), these enzymes have different catalytic mechanisms and belong to different gene families. Using a benchmarking strategy based on a permissive distance of two edges, would make no difference between glutathione peroxidase and catalase as their molecular functions are within the distance of two edges in the graph structure. Consequently, if in the starting test set there is a glutathione peroxidase which the method has predicted to be a catalase, this benchmarking strategy would erroneously assess this result as correct. For the same reasons, we have not considered semantic similarity as an appropriate evaluation measure of the benchmark results. Semantic similarity is a powerful technique for clustering putatively related functions, but is not necessarily an appropriate method for retrieving exact annotation as this strictly depends on the cut-off, the graph structure and the measure used [15], [21], [35].

### behavior and meaning of AC, InC and TS Indexes

To give an overall idea of the functional assignment performed by ARGOT we have developed three different and complementary indexes that may be useful in particular situations. Indeed, the three indexes behave in almost the same way although there is a slight prevalence of TS over the other two. The AC index is an absolute measure and gives an idea of how far the extracted GO term is from the maximal theoretical score. The lower the measure the further the extracted annotation is from the hypothetical ideal score, the latter being strictly dependent on how close or how far hits are related to the query. The InC index, on the other hand, is a conditional score that is calculated relative to all the retrieved hits and is independent of the method used to score the hits. Unlike AC, it does not depend on how the query relates to the found hits and is particularly effective in retrieving the more representative GO terms when hits share weak similarities with the query. The different behaviors of the two scores are shown in Fig. 3(c), 4(c) for AC and Fig. 3(e), 4(e) for InC. The AC trend is negatively affected by low sequence identity whereas InC is more stable, as expected. The net effect is that InC is more reliable with low scoring hits compared to AC as well as to TS (see Fig. 3(d), 4(d)). When using AC scores, the cut-offs must be chosen carefully taking into account how similar the hits are to the query (see cut-off ranges in Table 2). Finally, the TS index is statistically more robust as it considers how significant the GO term is using the Z-score of the weighted nodes, and in most cases is more reliable than AC and InC (see Fig. 3(a), 3(b), 4(a), 4(b)). In fact, the TS score is the InC multiplied by the Z-score and, given the stable trend of InC over different identity cut-offs, we expected TS to improve upon InC in any identity range or, at least, to behave similarly. The slight performance decrease is due to the fact that when only low scoring hits are retrieved by BLAST these are few and the rate of false positive alignments with unrelated sequences increases. In this particular condition, the Z-score proves to be ineffective and may negatively alter the InC index. The best cut-off ranges for annotating sequences with ARGOT are reported in Table 2.

**Table 2:** Results of the three indexes for the test sets RES and YEAST are reported.

YEAST 100	Cut-off	SENS	SPEC	PPV	NPV	ACC
**TS**	3	0.94	0.34	0.81	0.63	0.79
	5	0.84	0.64	0.88	0.56	0.79
**InC**	0.2	0.84	0.69	0.89	0.59	0.81
	0.3	0.72	0.82	0.93	0.48	0.74
**AC**	0.3	0.82	0.58	0.86	0.51	0.76
	0.4	0.75	0.67	0.88	0.46	0.73
**YEAST 40**						
**TS**	3	0.95	0.37	0.76	0.80	0.76
	5	0.83	0.65	0.83	0.66	0.77
**InC**	0.2	0.90	0.61	0.83	0.75	0.81
	0.3	0.78	0.74	0.86	0.63	0.77
**AC**	0.05	0.92	0.43	0.77	0.73	0.76
	0.1	0.86	0.59	0.81	0.67	0.77
**RES 100**						
**TS**	3	0.98	0.87	0.77	0.99	0.90
	5	0.95	0.90	0.81	0.98	0.92
**InC**	0.2	0.97	0.82	0.71	0.98	0.87
	0.3	0.90	0.85	0.73	0.95	0.86
**AC**	0.3	0.93	0.85	0.73	0.97	0.88
	0.4	0.89	0.88	0.76	0.95	0.88
**RES 40**						
**TS**	3	0.95	0.85	0.69	0.98	0.87
	5	0.88	0.89	0.74	0.95	0.89
**InC**	0.2	0.96	0.81	0.65	0.98	0.85
	0.3	0.90	0.86	0.71	0.96	0.87
**AC**	0.05	0.93	0.78	0.60	0.97	0.82
	0.1	0.84	0.85	0.67	0.93	0.85
**YEAST 4000**						
**TS**	3	0.93	0.35	0.83	0.60	0.80
	5	0.84	0.64	0.89	0.53	0.79
**InC**	0.2	0.84	0.69	0.90	0.55	0.80
	0.3	0.72	0.82	0.93	0.45	0.74
**AC**	0.3	0.84	0.55	0.87	0.49	0.77
	0.4	0.77	0.65	0.88	0.44	0.74
**Blast2GO**	default	-	-	0.71	-	-

Two cut-offs, chosen on the basis of more or less stringent criteria, are reported for each of the three scores. These thresholds represent the best trade-off found after ROC plot analyses. The cut-offs are 3, 5 for TS, 0.2, 0.3 for InC, 0.3, 0.4 for AC in RES 40 and YEAST 40 and 0.05, 0.01 for AC in YEAST 100, RES 100 and YEAST 4000. The first value represents the less stringent threshold. Sensitivity (SENS), specificity (SPEC), positive predicted value (PPV), negative predicted value (NPV), and accuracy (ACC) have been calculated for low sequence similarity hits below 40% identity (RES 40 and YEAST 40) and for high sequence similarity hits under 100% identity (RES 100 and YEAST 100). YEAST 4000 refers to the benchmarking subset of 4,000 yeast sequences used to make the comparison with Blast2GO performance (see text for further details).

### Test set RES

The results of test set RES are reported in Fig. 3 for ARGOT and TOPBLAST. Original GO annotations with UniProtKB sequence accession numbers and the results of ARGOT are supplied in the supporting material, Dataset S1 and Dataset S2 respectively. To check the robustness of ARGOT in predicting the correct function at different sequence similarities we have plotted the ROC100 in 3(a) with no restrictions applied to the sequence identity shared between query and hits. In 3(b) the ROC40 plot refers to over 700 sequences from our test set that share no more than 40% sequence identity with first BLAST hits. We made this distinction as most of the sequences of the test set share over 70% sequence identity with first BLAST hits and this could positively affect the assessment making functional inference an easy task. On the contrary, comparison of the curves obtained at different cut-offs and at 40% as shown in 3(b) has proven that ARGOT does not suffer evident performance loss and still shows high sensitivity without affecting specificity. What is unexpected is the great heterogeneity found in the annotations of the BLAST results used for assessing ARGOT. This is mainly due to a high false positive rate as shown in 3(f). The ROC plot is even more remarkable when the curve of the first BLAST hit is compared with those of the first two up to the first five hits. Unexpectedly again, the first hit does not seem to necessarily carry the correct information as, under our test conditions, its ROC curve is always below the others, although only slightly. In general, the overall trend of TOPBLAST curves is not far from the no discrimination line (the diagonal plotted line) and random guess. This proves that using a simple sequence similarity approach like BLAST for functional inference may not be so immediate nor trivial even at high sequence identity [37]. BLAST may be highly sensitive as the correct annotations can be extracted from the retrieved hits, but this occurs at the expense of having poor specificity due to the high number of false positives. As the main goal of every method is to have a good trade-off between false and true positives, ARGOT has been devised and tuned to take into account the potential false positive hits of whatever method is used, including BLAST, for functional annotation. This is accomplished by clustering the GO terms on the basis of their semantic similarity and ranking them. This allows ARGOT to outperform TOPBLAST as shown in Fig. 3, where the curves of the different scores lie near the upper left hand corner.

### Test set YEAST

The results of test set YEAST are reported in Fig. 4 for ARGOT and TOPBLAST. Original GO annotations with UniProtKB sequence accession numbers and the results of ARGOT are available in the supporting material, Dataset S3 and Dataset S4 respectively. Regarding the test set YEAST, the robustness of ARGOT was checked at different sequence similarities and we report in Fig. 4(a) the ROC100 plot, where no restrictions were applied to sequence identity between query and hits, and in 4(b) the ROC40 plot of over 600 sequences sharing no more than 40% sequence identity with first BLAST hits. As previously observed for the test set RES, the relative trends of the ROC curves demonstrate that ARGOT does not suffer evident performance loss and still shows high sensitivity without affecting specificity when sequence identity drops. Most of the conclusions and observations made with respect to test set RES apply just as well here, but some differences are worth mentioning. There is a general worsening of tool performance (see Table 2) given that the areas under the curves (AUC) are smaller than those of Fig. 3. Different factors contribute to this performance loss and one of them is certainly the elimination from UniProtKB of all the sequences from the Fungi kingdom that *S. cerevisiae* belongs to.

BLAST hits confirm that a higher ratio of distantly related proteins and fewer hits were found compared with test set RES (see Table 3). In addition, yeast is the most studied eukaryotic organism for which a huge amount of experimental data and highly detailed annotations, with an average of seven GO terms per protein, are available (see Fig. 2). These annotations are infrequent, even rare, in GOA and consequently difficult to retrieve. BLAST itself was able to recover only 44% of yeast GO annotations versus over 95% for the test set RES. The problem lies with the similarity search rather than with ARGOT as shown in Fig. 4(f), where BLAST is worse than in Fig. 3(f). In this case the ROC curves are closer to the diagonal of random guess and are almost straight lines. This confirms the presence of a higher number of false positives together with low coverage of correct GO terms in the BLAST results. Again, it is worth pointing out that the previously observed trend of the first BLAST hit is confirmed and constantly worse than in the other curves. In this case the tendency to consider more BLAST hits for functional inference seems to improve the chances of getting the correct annotation, the curve of the first five BLAST hits being the best (see “top 5 BLAST” in Fig. 4(f)). This is true only if first BLAST hits are taken into account since increasing their number lowers performance, as one might expect (data not shown).

**Table 3:** The figures represent the percentages of BLAST results with the first hit under different thresholds.

Identity	RES	YEAST
**40%**	7%	11%
**60%**	22%	38%
**80%**	43%	65%

The test set YEAST shows a higher percentage of low scoring hits compared to the test set RES.

### ARGOT versus Blast2GO

We compared ARGOT with Blast2GO over the same BLAST results (see supporting material Dataset S5) obtained for 4,000 proteins from the test set YEAST. The lack of a score associated to the retrieved GO annotations did not allow us to plot a ROC curve and we could not calculate false and true negatives. The only parameter we evaluated was PPV (see Table 2) and the value obtained was 0.71 whereas the worst ARGOT index was 0.83 for TS = 3 and the best 0.93 for InC = 0.3. Overall improvement, under our benchmark conditions, was not marginal with ranges from 15% to 25% depending on the cut-off and score used.

### Evaluation and manual inspection of the small reference test set

After the automatic procedure of extraction and manual check, 28 proteins satisfying our requirements (see M&M) were tested with six web tools (JAFA, GOTCHA, PhyDBAC, GOblet, PFP, InterProScan) and two local tools (ARGOT and Blast2GO). Performance of the external tools may be overestimated as they take advantage of the presence of the sequences themselves in protein databanks. For this reason, we have not discarded them from the BLAST results when assessing ARGOT and Blast2GO in direct comparison. In any case, the impact of their absence in the protein databank has been evaluated and reported in Table 4, Table 5 and supporting material Table S1 as “ARGOT W/O” (ARGOT without) and “Blast2GO W/O” (Blast2GO without). Whenever applicable, cut-offs were applied according to default values. GOblet was queried with an e-value of 10e^−1^, GOtcha with a 10% cut-off when used in JAFA and 30% when alone, and finally ARGOT with TS = 3. The complete results for “molecular function, “biological process” and “cellular components” produced by ARGOT are reported in the supporting material Table S2 for both “ARGOT” and “ARGOT W/O” tests.

**Table 4:** Results of the function prediction tools over a selected test set of 28 proteins.

tool	cut-off	results on 28 proteins	good	more specific	uncertain good	false positive	uncertain bad
**PFP**	default	**28**	1	1	**9**	**9**	**8**
**ARGOT**	TS = 3	**28**	**23**	**2**	2	-	1
**JAFA**	GOblet 10e^−01^, GOtcha 10%	**22**	**16**	-	2	1	**3**
**GOblet**	10e^−01^	**22**	**14**	-	1	**6**	1
**Blast2GO**	default	13	11	-	1	1	-
**InterProScan**	default	10	10	-	-	-	-
**GOtcha**	30%	5	2	1	2	-	-
**PhyDBAC**	default	2	1	-	-	1	-
**ARGOT W/O**	TS = 3	**22**	**17**	**2**	-	1	2
**Blast2GO W/O**	default	10	8	-	1	1	-

The reported data refer to annotation precision at the protein level: *i) good*, when all terms were either identical to the UniProtKB annotation, parental or related to the molecular function described for the same protein family; *ii) more specific*, if the annotation was *good* and one or more terms were child/children of the UniProtKB term; *iii) uncertain good*, when the first term was exact but others with high scores were false positives; *iv) false positive*, when all terms were not *good*; *v) uncertain bad*, if the first term was false positive and others had scores above the cut-off.

**Table 5:** Results of the function prediction tools over a selected test set of 28 proteins.

	total n. of GO terms	TP	related terms (RT)	more specific (MS)	FP	coverage % (TP+RT+MS)/62	PPV (TP+RT+MS)/(TP+RT+MS+FP)	cut-off
**Original annotation**	62							
**ARGOT**	52	**43**	**2**	4	4	**79.03**	**0.92**	3
**Blast2GO**	22	14	2	1	5	27.42	**0.77**	not applicable
**GOblet**	32	13	7	0	12	32.26	0.63	10e-1
**InterProScan**	12	11	1	0	0	19.35	**1.00**	not applicable
**GOtcha**	42	8	**25**	1	8	**54.84**	**0.81**	30%
**PhyDBAC**	4	0	2	0	2	3.23	0.50	not applicable
**JAFA**	38	**22**	7	2	7	**50.00**	**0.82**	not applicable
**PFP**	140	**21**	**19**	3	**97**	**69.35**	0.31	not applicable
**ARGOT W/O**	39	**25**	**6**	3	5	**54.84**	**0.87**	3
**Blast2GO W/O**	18	9	3	1	5	20.97	**0.72**	not applicable

The reported data refer to annotation precision at the GO level. The starting subset is composed of 62 annotations and predictions have been considered as *i) true positive* (TP) if the same as the original annotations, *ii) more specific* (MS) if a more precise functional description was obtained, *iii) related terms* (RT) if the prediction is either similar to or more generic than the original ones and *iv) false positive* (FP) if completely wrong.

Manual inspection allowed the meaning of correct annotation to be extended to close parent or child nodes and to give the reader accuracy at both protein (see Table 4) and GO levels (see Table 5) taking into account the “molecular function” ontology. The results were sorted into categories according to the match of the “best scoring hit” with the original UniProtKB annotation as follows (see Table 4): if no scores were available, as in the case of InterProScan and Blast2GO, all terms were considered equally. Table 5 reports the same results with evaluation of every single GO term prediction.

Four tools yielded results for more than 75% of the query proteins (PFP, JAFA, GOblet, and ARGOT) but ARGOT alone scored over 70% good results without erroneous annotations (Tables 4 and 5). In the worst case of “ARGOT W/O”, coverage at protein level was 78% and at GO level almost 55% with a PPV of 0.88 whereas in the best scenario it reached 100% and 79% coverage at the protein and GO levels respectively, with a PPV of 0.92 (see Tables 4 and 5). Blast2GO, InterProScan, and GOtcha scored >50% good annotations but only 20% to 50% of the queries produced results. The analysis performed after discarding the query protein from the databank demonstrates the efficiency of both ARGOT and Blast2GO tools despite lower coverage and an increase in false positives. In our test conditions, ARGOT performed better than the other tools, while JAFA, taking advantage of the joint predictions from GOblet, GOtcha, InterProScan and PhyDBAC, proved to be the best of the rest. Indeed, PhyDBAC was shown not to work properly considering that most of the proteins were from eukaryotic sequences and the tool had been specifically designed to predict the function of bacterial proteins from genomic context. On the other hand, the performance of GOtcha was affected by the chosen cut-off of 30%, suggested by the authors to avoid false positives [20] but negatively influencing coverage. Finally, PFP reached a high level of coverage and one more specific annotation but in our conditions it turned out to have a high false positive rate.

### Three example cases in detail

As mentioned above, manual inspection by expert users is of great value in exploring and assessing all of the possible functional features of a protein. Pre-processing and study of the 28 selected proteins revealed all of the expected limitations of automatic evaluation for large sets of sequences. Correct predictions would have been either rejected or considered false unless a careful manual inspection had been applied. We report here two different cases, originally annotated with generic GO terms, that some tools were able to refine in more detail and to catch inner functional features. These particular case study examples are listed in the supporting material Table S3. The first sequence, Q54EY2, is a subunit of the translation initiation factor (eIF-2B) which catalyzes the exchange of eukaryotic initiation factor 2-bound GDP for GTP [41] in *D. discoideum*. The original GO description defines its molecular function as a generic “GTP binding” property but the subunit was correctly assigned the more specific guanyl-nucleotide release factor activity by ARGOT, PFP and Blast2GO. This is an evident example of the incompleteness of annotations in databanks negatively affecting automatic evaluation of benchmark results as the two GO terms “guanyl-nucleotide exchange factor activity” (GO:0005085) and “GTP binding” (GO:0005525) are in different branches of the GO graph and do not share a parent. The distance between them is great, even with a semantic similarity approach. Any automatic assessment would inevitably evaluate this result as false positive. The second protein, Q9RC23, is the mersacidin decarboxylase (EC 4.1.1.-) from *Bacillus sp.*, a homodecameric lyase that catalyzes the oxidative decarboxylation of the C-terminal cysteine residue of mersacidin to an aminoenethiol residue [42]. Again, the UniProtKB databank reports only the generic GO terms “oxidoreductase activity”, “lyase activity” and “catalytic activity”, but experimental evidence suggests that this protein may possess a phosphopantothenoylcysteine decarboxylase activity or, at least, that the enzyme may belong to the protein family whose members have been demonstrated to catalyze this reaction. This result was obtained with ARGOT, Blast2GO, JAFA, GOtcha and PFP. The same conclusions as those drawn for the Q54EY2 protein when making an automatic functional assessment may be drawn here. On the other hand, the last example, protein O94436, is a transcription initiation factor TFIID subunit 14 [43] which was erroneously predicted as a translation factor by ARGOT. This is due to the fact that first scoring hits (UniProtKB accession numbers are: A1CBG5, A1DDX4, Q2U688, Q6M9J3, A5DUJ4, Q4WVI2) were erroneously annotated as translation factors with GO terms belonging to the IEA category (as of March 2008). These proteins are, in fact, transcription factors but in this particular case the reported IEA annotations are incorrect. Both the biological process and the cellular component of these hits properly refer to their transcription factor activity and complexity respectively. Indeed, ARGOT assigned the correct biological process and cellular component as expected (see supporting material Table S2). This is a clear example of how IEA invalid annotations, being completely automatic, can propagate in the databanks.

## Discussion

The present work deals with the implementation of a fast annotation system which is able to cluster GO terms on the basis of their semantic similarity and their calculated weights. The grouping of these clusters allows for detection of highly populated areas of the graph and significant representatives are chosen based on their information content and weight that take into account how biologically similar they are to the query. The latter information may be acquired by simply performing a similarity search using BLAST, as we have shown here for benchmarking.

To our knowledge, this is the first integrated approach that implements both a weighting scheme and semantic similarity to select the correct annotation. This approach has proven to be more effective than other methods, especially TOPBLAST. We based our test on simple BLAST results, and it may be argued that this sounds neither attractive nor novel. We partly agree with this view, but it must be borne in mind that the majority of the most sophisticated methods developed so far still rely on different strategies based on similarity searches, profiles, etc. for functional inference [4]. Moreover, it is common practice to use BLAST or other similarity search tools to query protein databanks first [44]. Furthermore, fresh data and updates to new and old protein records are usually submitted to primary repositories such as UniProtKB, but this knowledge takes time to propagate and to become available in specialized databases, such as those consisting of protein domains. The only way to get immediate access to this information is by querying primary databases using a similarity search. Unfortunately, other deficiencies affect databanks and submitted sequences usually lack annotations in the form of GO terms or may be incomplete or obsolete [17]. The GOA consortium is making a considerable and timely effort to rectify these deficiencies and some improvement has already been made. Having easy access to the source of raw data may, in any case, make a difference. ARGOT exploits directly this primary information and, moreover, has been designed to be independent of BLAST in future releases. This will allow us to overcome the present limitations of BLAST searches, which suffer from various defects [45] such as: *i)* those concerning the local alignment strategy when dealing with multiple domain proteins, *ii)* the inability to find distantly related sequences sharing low or no similarity but having the same function, *iii)* the false positive rate that we and other authors have demonstrated to be high [37], *iv)* excessive transfer of annotations, especially when dealing with paralogs versus orthologs [3].

Another important question that still remains unanswered is the design of an acknowledged standard to perform rigorous and easy comparable benchmarks [12], [13], [46]. In addition, the frequent changes and updates in both the Gene Ontology graph structure and the GOA database of annotations need to be traced, as changes may affect true comparisons between methods developed and assessed over different benchmark test sets at different times [40], [47]. There is an open debate and worth attempts have been proposed as the AFP endeavor [48]. The issue is indeed a thorny one as there is not even agreement over the definition of protein function, one of the most elusive concepts in biology for the reasons mentioned in the introduction and, more importantly, there are still few and incomplete experimental data to be efficiently exploited [13]. This is evident in the restricted and well-studied set of 28 proteins which lack certain valuable GO annotations. This incomplete and fragmented knowledge impacts the false positives rate as shown by the analyses of the reported cases. Indeed, the real dimension of this phenomenon is difficult to quantify, but it may limit and affect rigorous evaluation of an automatic, comprehensive, and extended blind test over thousands of proteins. Any solution may be flawed and manual intervention is not viable especially when dealing with large sets of sequences. This is why we decided not to complicate the function prediction benchmark. We used a plain and conservative approach where only the recovery of exact original annotations was taken into account, to avoid bias or overestimation of tool performance. Even though this is a rough measure and a controversial approach, it is certainly one of the best alternatives to an unfeasible manual control over all the sequences of the test set. In any case, the more sequences that are tested the more the intrinsic biases and defects of the benchmarking should become statistically marginal.

Finally, ARGOT has proven to be a fast and precise tool whose main goal is to manage easily thousands of proteins in a typical large scale genome project [28], but can also be effective on a small scale and for single protein annotations, in order to gain general insights into putative functions [29]. In the former case, the updating of many thousands of sequences, whose BLAST results have been acquired already, is a matter of a few hours on an ordinary desktop computer and can be easily performed on a weekly basis. This allows for comparison between the different annotation releases to be made in order to trace differences over a given time-span and for the functional annotations to be kept updated. In the latter case, we have demonstrated the efficiency of the ARGOT tool over a small set of 28 sequences with particular annotations and low sequence similarities with known proteins. Its levels of sensitivity and specificity are, in fact, encouraging reaching values of 0.95 and 0.87 respectively using the TS index (TS = 3 for RES 100 test set). When there is low sequence similarity the InC index (InC = 0.3 for RES 40 test set) still shows a high degree of confidence with 0.90 sensitivity and 0.86 specificity. These data were confirmed in the small test set of 28 proteins where accurate manual investigation allowed the intrinsic limitations of automatic assessment to be bypassed.

Future developments are planned to be able to implement different sources of data, such as the integration of protein domain profile searches and specialized protein databanks annotated with GO, in order to improve the functional coverage and predictive power tested over BLAST and UniProtKB.

## Supporting Information

Dataset S1 original GO annotations of the test set RES
(0.79 MB TXT)

Dataset S2 ARGOT results for test set RES
(5.24 MB TXT)

Dataset S3 original GO annotations of the test set YEAST
(0.77 MB TXT)

Dataset S4 ARGOT results for test set YEAST
(1.23 MB TXT)

Dataset S5 Blast2GO results obtained for the benchmark test set of 4000 Yeast proteins
(0.30 MB TXT)

Table S1 The table reports the results obtained from different function prediction web servers for the small test set of 28 proteins
(0.07 MB XLS)

Table S2 Complete results for “molecular function, “biological process” and “cellular components” produced by ARGOT for both “ARGOT” and “ARGOT W/O” tests
(0.11 MB XLS)

Table S3 Prediction data from the tested tools for three particular cases
(0.03 MB XLS)
